# Acute Poisoning With Datura stramonium Plant Seeds in Qatar

**DOI:** 10.7759/cureus.20152

**Published:** 2021-12-04

**Authors:** Ahmed Shebani, Mohamed Hnish, Hussam Elmelliti, Muhnad Mohamed Abdeen, Adel Ganaw

**Affiliations:** 1 Internal Medicine, Hamad Medical Corporation, Doha, QAT; 2 Family Medicine, Hamad Medical Corporation, Doha, QAT; 3 Emergency Medicine, Hamad Medical Corporation, Doha, QAT; 4 Critical Care Medicine, Hamad Medical Corporation, Doha, QAT

**Keywords:** substance abuse, toxic delirium, anticholinergic syndrome, jimson weed, datura stramonium

## Abstract

*Datura stramonium* (also called thorn apple or Jimson weed) is a plant that contains atropine, scopolamine, and hyoscyamine, giving it anticholinergic effects when consumed. We report the case of a 32-year-old male in Qatar who intentionally ingested seeds from Jimson weed mixed with milk. The patient became severely confused and delirious, eventually requiring admission to the intensive care unit (ICU) for two days for management. The patient was discharged safely with no complications afterward. The case is unique in that Jimson weed is not common in Qatar, and due to the adverse effects of this plant, this case serves to highlight to both the general population and healthcare professionals the effects of ingestion and the appropriate management plan for toxicity caused by Jimson weed.

## Introduction

*Datura stramonium* (commonly known as thorn apple or Jimson weed) is a plant used by some people as a recreational drug, citing a heightened sense of well-being or euphoria. When ingested, it activates the anticholinergic system because it contains atropine, scopolamine, and hyoscyamine [1]. The seeds contain the highest concentration of toxic agents, with approximately 0.1 mg of atropine per seed or 3-6 mg per 50-100 seeds [2].

In general, Jimson weed toxicity symptoms resemble atropine poisoning, including dryness of the mouth and skin, extreme thirst, pupil dilation with impaired vision, urinary retention, tachycardia, confusion, restlessness, hallucinations, and loss of consciousness [3]. These toxic manifestations start to appear one to four hours post-ingestion and may last for 24-48 hours [4].

We present a rare case of intentional ingestion of Jimson weed seeds mixed with milk by a 32-year-old male in Qatar, a region where Jimson weed is uncommon, who became confused and delirious and was treated in the intensive care unit (ICU) for the plant's anticholinergic adverse effects. While there were two reported cases of Jimson weed intoxication in Saudi Arabia in 1984 and 2015, where the plant grows widely in Saudi Arabia's rural areas [5,6], this case marks the first instance of Jimson weed intoxication in Qatar.

## Case presentation

**Figure 1:**
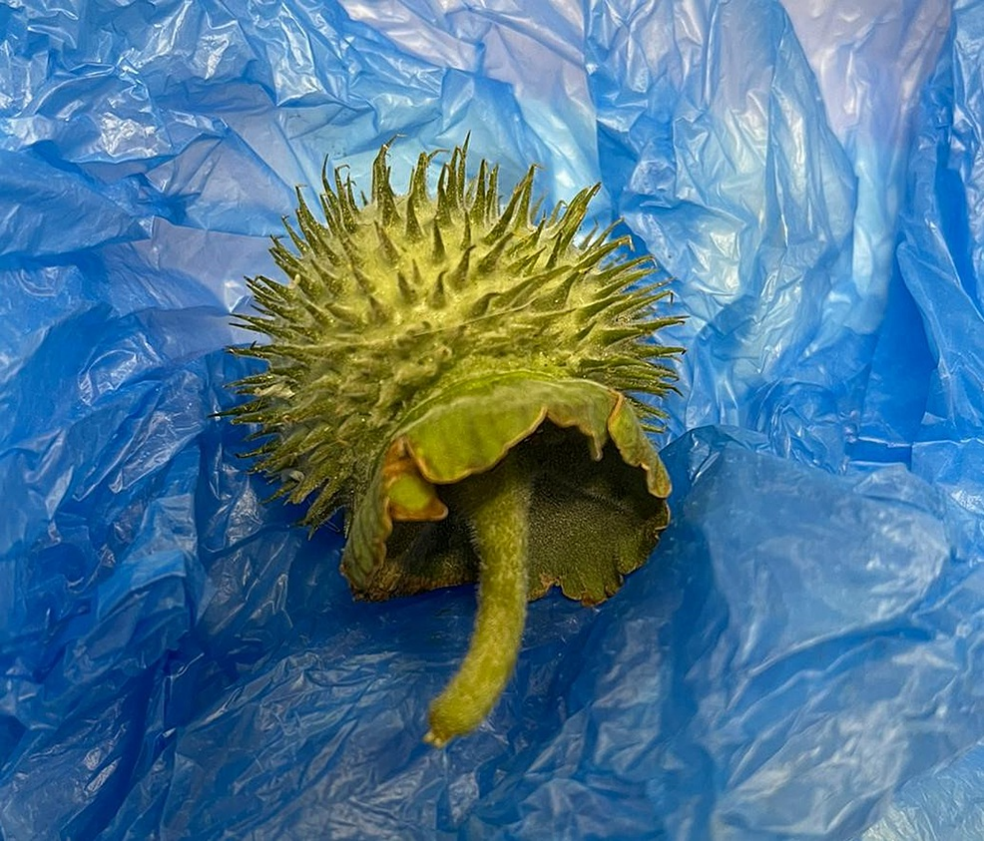
Jimson weed plant

A 32-year-old Nepalese male who lives in Qatar with no significant past medical history was brought to the emergency department (ED). He presented with confusion, delirium, and agitation. The patient showed aggressive behavior and was unable to communicate. His family reported that he had ingested Jimson weed seeds boiled in milk three to four hours prior to arrival at the ED, and they brought the plant with them to the ED (Figure 1).

**Figure 2:**
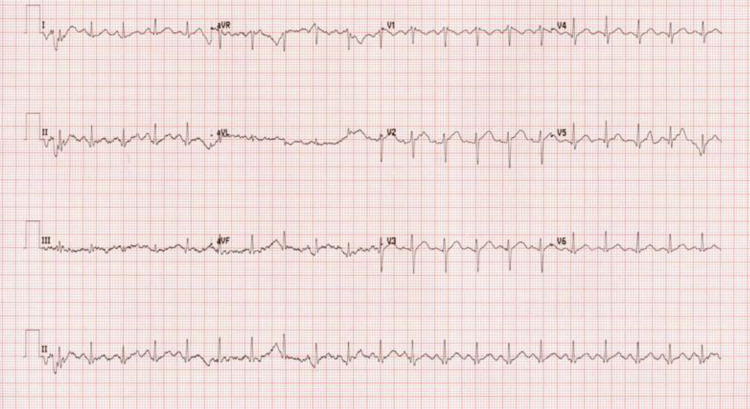
ECG

On presentation, the patient was vitally stable, except for sinus tachycardia noted via electrocardiogram (ECG) (Figure 2), with a heart rate of 118 beats per minute, body temperature of 36.6°C, respiratory rate of 22 breaths per minute, blood pressure of 149/85 mmHg, and oxygen saturation of 96% on room air. On examination, we noted the patient had dry, flushed skin with dilated pupils. He was confused and delirious and could not communicate. He had a Glasgow Coma Scale (GCS) score of 10/15, and power and tone were normal in all limbs. The findings of his cardiovascular, respiratory, gastrointestinal, and genitourinary system examinations were all unremarkable.

At the time of admission, we ordered laboratory investigations including complete blood count, urea, creatinine and electrolytes, liver function test, creatine kinase level, random glucose level, myoglobin level, ethanol level, and arterial blood gas; the results of all laboratory investigations were within reference ranges; however, the patient had mildly elevated alanine transaminase (49 U/L) and elevated myoglobin levels (92 ng/mL). On the second day of hospitalization, the second set of laboratory investigations were conducted in addition to a coagulation profile; all results were within reference ranges, except for elevated myoglobin levels (252 ng/mL) (Table 1).

**Table 1:** Laboratory investigation results

Analyte	Reference Range	At Admission	Day 1 After Admission	On Discharge Day
WBC (µL)	4–10	10.8	13	9.7
Hgb (g/dL)	13–17	16.4	16.2	16.3
Hct (%)	40–50	46.5	47	48
Plt (µL)	150–400	349	322	324
Glucose (mmol/L)	3.3–5.5	6.7	5.6	5.8
Urea (mmol/L)	2.5–7.8	3.9	3	3.6
Creatinine (µmol/L)	62–106	83	86	86
Sodium (mmol/L)	135–145	137	139	139
Potassium (mmol/L)	3.5–5.3	3.9	4	4.3
Bilirubin T (µmol/L)	0–21	8	12	22
ALP (U/L)	40–129	62	57	61
AST (U/L)	0–40	30	28	28
ALT (U/L)	0–40	49	40	42
CK (U/L)	39–308	153	NA	NA
Myoglobin (ng/mL)	28–72	92	252	36

The toxicology team reviewed the patient and suspected anticholinergic activity due to the ingestion of Jimson weed. They advised conservative management and observation in the ICU. The patient was treated conservatively with full seizure precaution measures, and he required benzodiazepines 20 mg total (lorazepam 10 mg intramuscular (IM) and midazolam 10 mg IM) for agitation. Activated charcoal was withheld due to his low GCS and his agitated state. After two days of ICU admission, the patient returned to his usual state; he was vitally stable; fully oriented to time, place, and person; and discharged home.

The Institutional Review Board (IRB) at Hamad Medical Corporation (HMC) and Medical Research Council (MRC) provided ethical approval to publish this case report (MRC 04-21-457). The patient provided written informed consent for his anonymized information to be published in this article.

## Discussion

*Datura stramonium* (i.e., Jimson weed) is an alkaloid plant containing anticholinergic substances, including atropine, which leads to the inhibition of central and peripheral muscarinic neurotransmission. For centuries, Jimson weed seeds have been used as a recreational drug for their hallucinogenic and euphoric effects [7].

The known symptoms of *Datura stramonium* intoxication include dry skin and mucosa, flushing, mydriasis, and sinus tachycardia, which are evident in our case and well known as the earliest and most reliable sign of anticholinergic toxicity. Additional clinical manifestations include decreased bowel activity, urinary retention, and hyperthermia, leading to rhabdomyolysis and multiorgan failure, such as liver, kidney, and brain damage. Several neurological manifestations occur, including ataxia, impaired short-term memory, disorientation, confusion, hallucinations (visual and auditory), psychosis, agitated delirium, seizures, and coma [7].

Our patient intentionally ingested the plant seeds for the euphoric and hallucinogenic effects. Although the plant does not grow naturally in Qatar, the patient confirmed that he grew the plant by himself to use its seeds. This is the first case reported in Qatar, but several cases had similar clinical presentations in nearby Saudi Arabia [5].

The diagnosis of anticholinergic intoxication is mainly clinical, and in our index patient, the family brought the plant, so there was no need for toxin serum workup. To exclude other co-ingestions and hypoglycemia, we investigated ethanol and serum glucose levels, but the results were within reference ranges. Cardiovascular collapse and respiratory failure can occur in severe cases [2], but in our patient, other than tachycardia, his blood pressure and respiration pattern were stable throughout his admission. Rhabdomyolysis and severe hepatic failure have been reported in the literature [8].

One of the effects of anticholinergic toxicity is the disturbance of the heart's electrical conduction, so an ECG is crucial to rule out any dysrhythmia and calculate the QRS interval duration. Our patient's ECG showed sinus tachycardia with QTc at 388 ms, and repeated ECG during hospitalization days showed no significant abnormalities.

The management of Jimson weed intoxication is mainly supportive and symptomatic, including monitoring vital signs and urine output and maintaining patent airways and circulation. The role of physostigmine in anticholinergic toxicity is controversial and reserved only for cases that show severe peripheral and central anticholinergic toxicity manifestations [7]. Physostigmine usually needs toxicology team approval before its use; in this scenario, the toxicology team recommended supportive measures only.

The effects of anticholinergic activity may last for 24-48 hours, which requires observation until complete resolution of symptoms, which can occur within six hours in mild cases or longer in moderate to severe cases. Our patient stayed in the ICU for 48 hours to achieve full recovery prior to discharge.

## Conclusions

*Datura stramonium* (i.e., Jimson weed) is sometimes used recreationally for its hallucinogenic and euphoric effects. It has anticholinergic effects and may cause neurological manifestations and multiorgan failure. Jimson weed is not common in Qatar, and to the best of our knowledge, this is the first case to be reported in Qatar. It shines a light on a new type of recreational material and its serious complications, which sometimes may necessitate ICU admission. Local awareness may be needed for populations and practitioners.
